# 2290: Control of atherosclerosis regression by LXRα S198 phosphorylation

**DOI:** 10.1017/cts.2017.34

**Published:** 2018-05-10

**Authors:** Elina Shrestha, Maud Voisin, Tessa J. Barrett, Hitoo Nishi, Inés Pineda-Torra, Edward A. Fisher, Michael J. Garabedian

## Abstract

OBJECTIVES/SPECIFIC AIMS: Accumulation of cholesterol-laden macrophages in arterial walls leads to atherosclerosis. LXRs induce expression of genes that are atheroprotective in macrophages including CCR7, a chemokine receptor that promotes their emigration from the plaque. CCR7 expression has been shown to be negatively regulated by phosphorylation of LXRα at S198 and is reduced in diabetic mice that show impaired plaque regression. I hypothesized that LXRα phosphorylation at S198 diminishes macrophage emigration from atherosclerotic plaque and contributes to impaired regression in diabetes. METHODS/STUDY POPULATION: Inducible LXRα S198A phosphorylation deficient knock in mouse were used as donors for bone marrow transplantation into mice prone to develop atherosclerosis. Plaques were developed by placing mice on western diet; and regression was induced by lowering their lipid levels. Aortic plaques were then analyzed by using morphometric, histological, and molecular analyses in control and diabetic mice expressing either LXRα WT or LXRα S198A during regression. RESULTS/ANTICIPATED RESULTS: Surprisingly, lack of phosphorylation increased plaque macrophage content and impaired regression under normoglycemic condition; however, it did not exacerbate diabetic regression. Plaques in diabetic mice were associated with increased LXRα S198 phosphorylation. Consistent with this, LXRα phosphorylation is enhanced in macrophages cultured under hyperglycemic conditions indicating glucose-dependent regulation of LXRα phosphorylation. Monocyte trafficking studies reveal that lack of phosphorylation and diabetes independently increase recruitment of monocytes in the plaque that might contribute to increased macrophage content. Importantly, I found that diabetes also increases macrophage retention in the plaque, which is reversed in the absence of phosphorylation. We predict that this increased retention results from inhibition of emigration of plaque macrophages through enhanced phosphorylation in diabetes. DISCUSSION/SIGNIFICANCE OF IMPACT: These findings suggest that inhibiting LXRα phosphorylation could be beneficial in diabetic atherosclerosis to reverse the accumulation of macrophages in the plaque. This study imparts insight on regulation of plaque macrophage trafficking through LXRα S198 phosphorylation.

